# Effector Vγ9Vδ2 T cell response to congenital *Toxoplasma gondii* infection

**DOI:** 10.1172/jci.insight.138066

**Published:** 2021-08-23

**Authors:** Ling Ma, Maria Papadopoulou, Martin Taton, Francesca Genco, Arnaud Marchant, Valeria Meroni, David Vermijlen

**Affiliations:** 1 Department of Pharmacotherapy and Pharmaceutics,; 2 Institute for Medical Immunology, and; 3 ULB Center for Research in Immunology, Université Libre de Bruxelles (ULB), Brussels, Belgium.; 4 IRCCS San Matteo Polyclinic, Pavia, Italy.; 5 Molecular Medicine Department, University of Pavia, Italy.

**Keywords:** Immunology, Infectious disease, Adaptive immunity, T cell receptor, T cells

## Abstract

A major **γδ** T cell population in human adult blood are the V**γ**9V**δ**2 T cells that are activated and expanded in a TCR-dependent manner by microbe-derived and endogenously derived phosphorylated prenyl metabolites (phosphoantigens). V**γ**9V**δ**2 T cells are also abundant in human fetal peripheral blood, but compared with their adult counterparts they have a distinct developmental origin, are hyporesponsive toward in vitro phosphoantigen exposure, and do not possess a cytotoxic effector phenotype. In order to obtain insight into the role of V**γ**9V**δ**2 T cells in the human fetus, we investigated their response to in utero infection with the phosphoantigen-producing parasite *Toxoplasma gondii* (*T*. *gondii*). V**γ**9V**δ**2 T cells expanded strongly when faced with congenital *T*. *gondii* infection, which was associated with differentiation toward potent cytotoxic effector cells. The V**γ**9V**δ**2 T cell expansion in utero resulted in a fetal footprint with public germline-encoded clonotypes in the V**γ**9V**δ**2 TCR repertoire 2 months after birth. Overall, our data indicate that the human fetus, from early gestation onward, possesses public V**γ**9V**δ**2 T cells that acquire effector functions following parasite infections.

## Graphical abstract

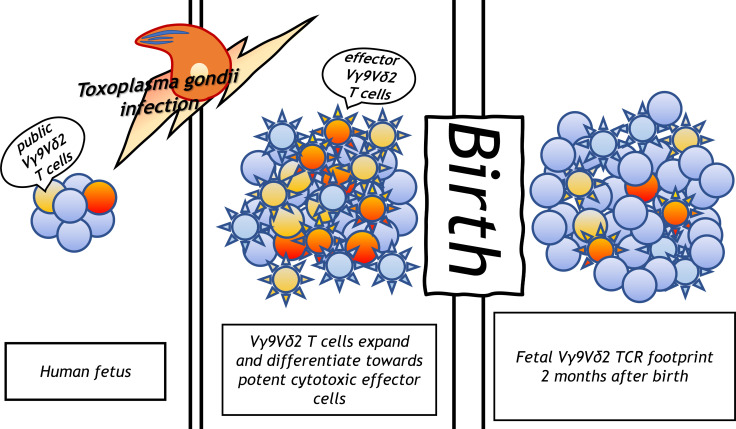

## Introduction

γδ T cells are T lymphocytes that express a TCR containing γ and δ chains, instead of α and β chains as in the conventional CD4^+^ and CD8^+^ αβ T cells. As αβ T cells, γδ T cells have been conserved for more than 450 million years of evolution and play an important role against infection and cancer (1–3). A major γδ T cell population in human adult blood are the Vγ9Vδ2 T cells that are defined by the expression of a TCR containing the γ chain variable region 9 (Vγ9, TRGV9) and the δ chain V region 2 (Vδ2, TRDV2). They express a potent cytotoxic effector phenotype and are activated and expanded in a TCR-dependent manner by microbe- and host-derived phosphorylated prenyl metabolites (phosphorylated Ags, or phosphoantigens), derived from the isoprenoid metabolic pathway (4–6). The prototypical example of a microbial phosphoantigen is (E)-4-hydroxy-3-methyl-but-2-enyl pyrophosphate (HMBPP) produced by the 2-Cmethyl-d-erythritol 4-phosphate (MEP) pathway of isoprenoid synthesis (also known as the nonmevalonate pathway) that is present in bacteria and in protozoan parasites of the phylum Apicomplexa (4). The recognition of phosphoantigens allows adult Vγ9Vδ2 T cells to develop potent antimicrobial and anticancer immune responses (2, 4, 7–9). While Vγ9Vδ2 T cells are also abundant in fetal peripheral blood, they are hyporesponsive toward phosphoantigen stimulation in vitro, they are highly regulated by programmed cell death protein 1, and they do not show a cytotoxic effector phenotype (10–15). These features are likely related to (tolerance) requirements of the fetal immune system, which involves a distinct thymic development (16–18).

During development of T cells in the thymus, TCR gene rearrangements take place where single V (variable), D (diversity; only for TRD), and J (joining) gene segments join to form a final chain (α, β, γ, or δ). The variability created during the V(D)J recombination is significantly enhanced by the junctional diversity, which comprises (a) incorporation of palindromic sequences (“P nucleotides”), (b) the introduction of additional random nucleotides (“N additions”) in the junction by the terminal deoxynucleotidyl transferase (TdT enzyme), and (c) deletion of nucleotides (by exonuclease) (19). The pairing of a single γ (TRG) with a δ (TRD) chain will give rise to the final TCR expressed on the surface of the γδ T cell. The most variable domain, usually responsible for antigen recognition, is found in the complementarity determining region 3 (CDR3) and is the region most often analyzed. In human, γδ T cells are the most abundant lymphoid population in the embryonic thymus in early gestation, with a shift around gestation week 11, when they decrease significantly and the αβ T cells take the lead (20). The very first γδ T cell population to arise in humans is the Vγ9Vδ2 subset, detected in the embryonic (prethymic) liver from as early as 5 to 6 weeks of gestation (21) and in fetal thymus after 8 weeks of gestation (17, 22), which is then likely to exit the thymus toward the fetal blood (10, 19). We have recently shown that fetal and adult Vγ9Vδ2 T cells are generated by the thymus at different time points in life, and we have identified key differences between the TCR repertoire of fetal and adult blood Vγ9Vδ2 T cells, including the very low number of N additions in the fetal Vγ9Vδ2 TCR repertoire (17).

*Toxoplasma gondii* (*T. gondii*) is an obligate intracellular protozoan that belongs to the phylum Apicomplexa and, thus, produces the potent phosphoantigen HMBPP. It infects up to one-third of the world’s population, and infection acquired during pregnancy (congenital infection) may cause severe damage to the fetus (23, 24). Previous studies indicate that γδ T cells play a role in the immune response against *T*. *gondii*. Indeed, mice depleted of γδ T cells are more susceptible to *T*. *gondii* infection (25), human adult Vγ9Vδ2 T cells are expanded upon acute toxoplasmosis, and an in vitro study showed that Vγ9Vδ2 T cells are activated by incubation with *T*. *gondii*–infected cells, resulting in the killing of the infected cells (26). While some changes have been observed in cord blood Vγ9Vδ2 T cells in placental malaria (27), it remains unclear whether fetal Vγ9Vδ2 T cells can develop immune responses against pathogens that cross through the placenta, such as in congenital toxoplasmosis (13, 15, 28, 29). We found that fetal Vγ9Vδ2 T cells expanded strongly and differentiated toward potent killer effector cells in infants with congenital *T*. *gondii* infection.

## Results

### Study population.

**Figure 1:**
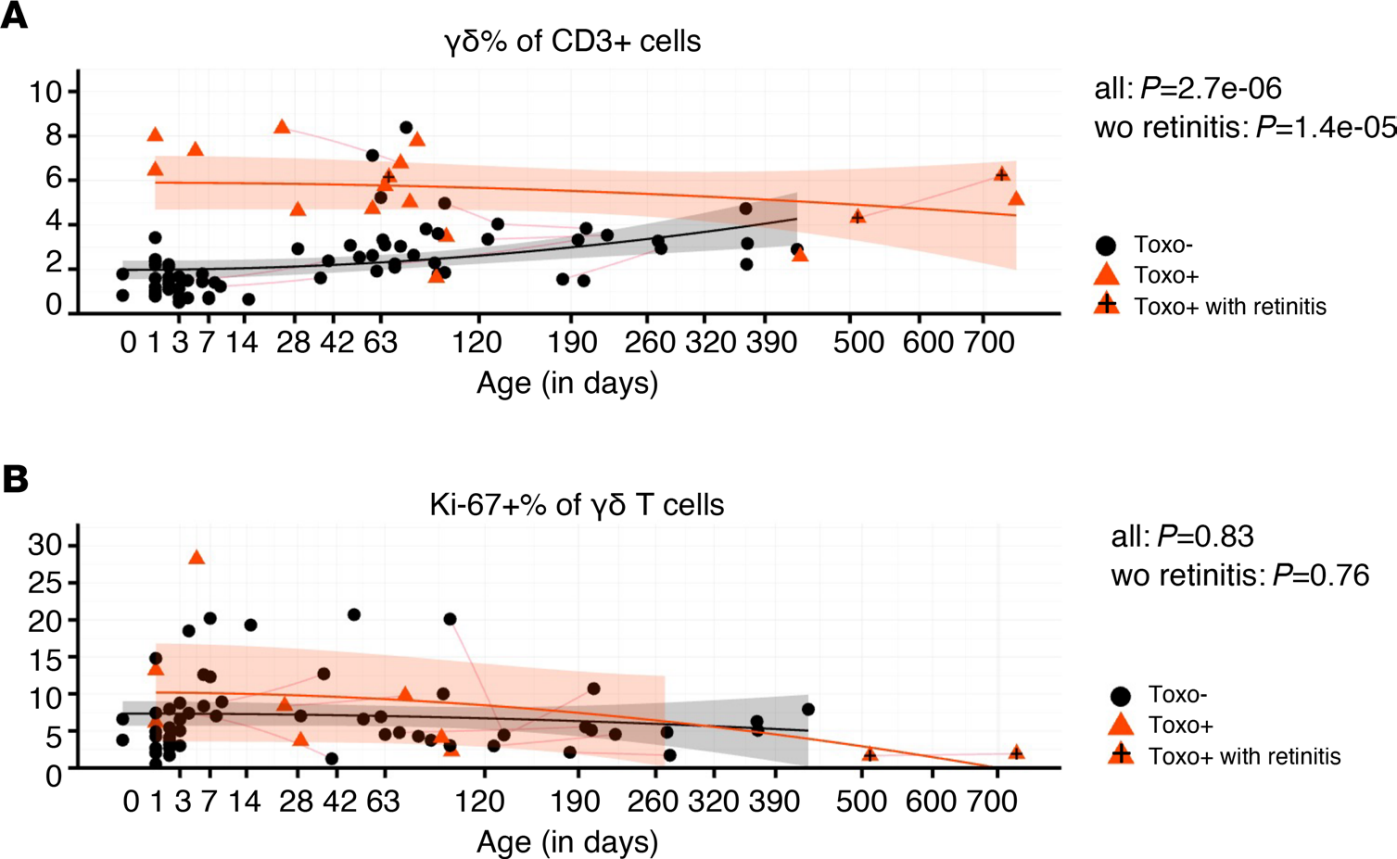
Congenital *T. gondii* infection induces expansion of γδ T cells in utero. (**A**) Percentage of γδ T cells (of total CD3^+^ T cells) versus age (sample number: Toxo^–^: *n* = 66, Toxo^+^: *n* = 17; subject number: *n* = 74, Toxo^+^ *n* = 15). (**B**) Expression of the proliferation marker Ki-67 in γδ T cells versus age (sample number: Toxo^–^: *n* = 55, Toxo^+^: *n* = 10; subject number: *n* = 56, Toxo^+^ *n* = 8). Toxo^+^ samples are indicated in orange triangles; Toxo^–^ samples are indicated in black dots. Lines connect samples of the same subject. Samples from subjects with symptoms (retinitis) are indicated with plus signs. *P* values of Toxo^+^ (with or without the subjects with symptoms) samples versus Toxo^–^ samples are calculated by Mann-Whitney *U* test. The 95% confidence interval (linear model) of more than 0 area is indicated for each group (orange for Toxo^+^, gray for Toxo^–^).****

Pregnant mothers with primary *T*. *gondii* infection were enrolled in this study. In order to address the fetal/newborn immune response toward *T*. *gondii* infection, blood samples from their infants were collected. Fifteen out of 74 infants (20%) were diagnosed with congenital toxoplasmosis (Toxo^+^). The Toxo^+^ infants were age matched with their noninfected (Toxo^–^) counterparts (Figure 1A; age of Toxo^–^ versus Toxo^+^ subjects: *P* = 0.0688). For most infants (66 out of 74), 1 blood sample was collected, for 7 infants 2 blood samples, and for 1 infant 3 blood samples were collected between birth and 2 years of age (Figure 1A). Clinical characteristics, such as age at the moment of diagnosis and treatment schedules of the Toxo^+^ infants, can be found in Table 1. All deliveries (of Toxo^+^ and Toxo^–^ infants) were term deliveries (>37 weeks gestation). No clinical problems were observed in the Toxo^–^ infants.

### Vγ9Vδ2 T cells expand strongly upon congenital T. gondii infection.

First, we determined the percentage of γδ T cells (out of total T cells) and found that they were significantly increased in Toxo^+^ infants (Figure 1A). As can be seen from the plotting of the percentage of γδ T cells versus the age of the infants, this higher percentage of γδ T cells was especially evident early after birth (0- to 2-month-old newborns, Figure 1A). At older ages (>2 months), the difference between Toxo^+^ and Toxo^–^ infants diminished, mainly because the percentage of γδ T cells started to increase in the Toxo^–^ group (Figure 1A). The increase in γδ T cells’ percentages early after birth was not associated with an increased expression of the proliferation marker Ki-67 (Figure 1B). To ascertain that this high percentage of γδ T cells was not due to changes in αβ T cells, we quantified absolute numbers and confirmed that the γδ T cells were increased, while no change could be detected in the αβ T cell compartment (Supplemental Figure 1, A and B; supplemental material available online with this article; https://doi.org/10.1172/jci.insight.138066DS1).

**Figure 2:**
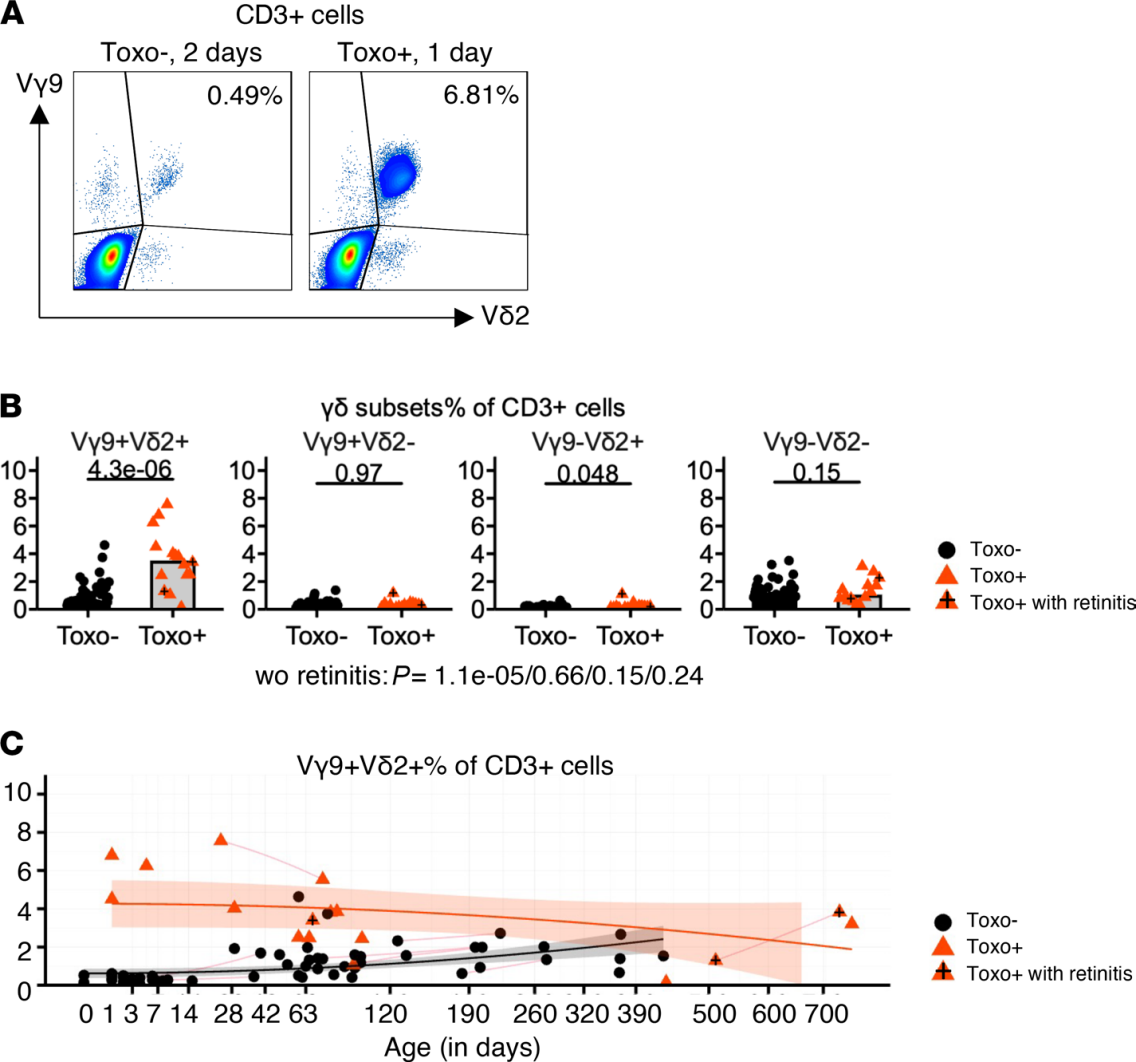
The expansion of γδ T cells upon congenital *T. gondii* infection is highly restricted to Vγ9^+^Vδ2^+^ T cells. (**A**) Representative flow cytometry plots of a Toxo^–^ (2 days old) and a Toxo^+^ (1 day old) newborn. Gate is on CD3^+^ T lymphocytes; percentage of Vγ9^+^Vδ2^+^ cells are indicated. (**B**) γδ Subsets’ percentage (of CD3^+^ T cells) (sample number: Toxo^–^: *n* = 66, Toxo^+^: *n* = 17; subject number: *n* = 74, Toxo^+^ *n* = 15). (**C**) Percentage of Vγ9^+^Vδ2^+^ cells (of total CD3^+^ T cells) versus age (sample number: Toxo^–^: *n* = 66, Toxo^+^: *n* = 17; subject number: *n* = 74, Toxo^+^ *n* = 15). Toxo^+^ samples are indicated in orange triangles; Toxo^–^ samples are indicated in black dots. Lines connect samples of the same subject. Samples from subjects with symptoms (retinitis) are indicated with plus signs. *P* values of Toxo^+^ (with or without the subjects with symptoms) samples versus Toxo^–^ samples are calculated by Mann-Whitney *U* test. The 95% confidence interval (linear model) of more than 0 area is indicated for each group (orange for Toxo^+^, gray for Toxo^–^).

Next, we investigated the Vγ9Vδ2 subset more specifically and compared it with other γδ subsets. Using antibodies specifically against the Vγ9 and Vδ2 chain (combined with CD3 and pan-γδ antibodies), we could delineate 4 different populations: Vγ9^+^Vδ2^+^, Vγ9^+^Vδ2^–^, Vγ9^–^Vδ2^+^, and Vγ9^–^Vδ2^–^ γδ T cells (Figure 2, Supplemental Figure 1C, and Supplemental Figure 2). The increase in newborn γδ T cells upon congenital *T*. *gondii* infection was highly restricted to the Vγ9^+^Vδ2^+^ subset (Figure 2, Supplemental Figure 1C, and Supplemental Figure 2); the other subsets did not show different percentages or numbers compared to Toxo^–^ newborns, including the more abundant Vγ9^–^Vδ2^–^ γδ T cell subset (Figure 2B, Supplemental Figure 1C, and Supplemental Figure 2). Gating on newborn Vγ9Vδ2 T cells, again no increase in the Ki-67 proliferation marker could be observed between Toxo^+^ and Toxo^–^ subjects (Supplemental Figure 3), indicating that the proliferation of Vγ9Vδ2 T cells had already occurred in utero, accounting for the higher percentage and number of newborn Vγ9Vδ2 T cells in the Toxo^+^ compared with the Toxo^–^ group (Figure 2, Supplemental Figure 1C, and Supplemental Figure 2).

### Newborn Vγ9Vδ2 T cells are highly differentiated upon congenital *T. gondii* infection.

**Figure 3:**
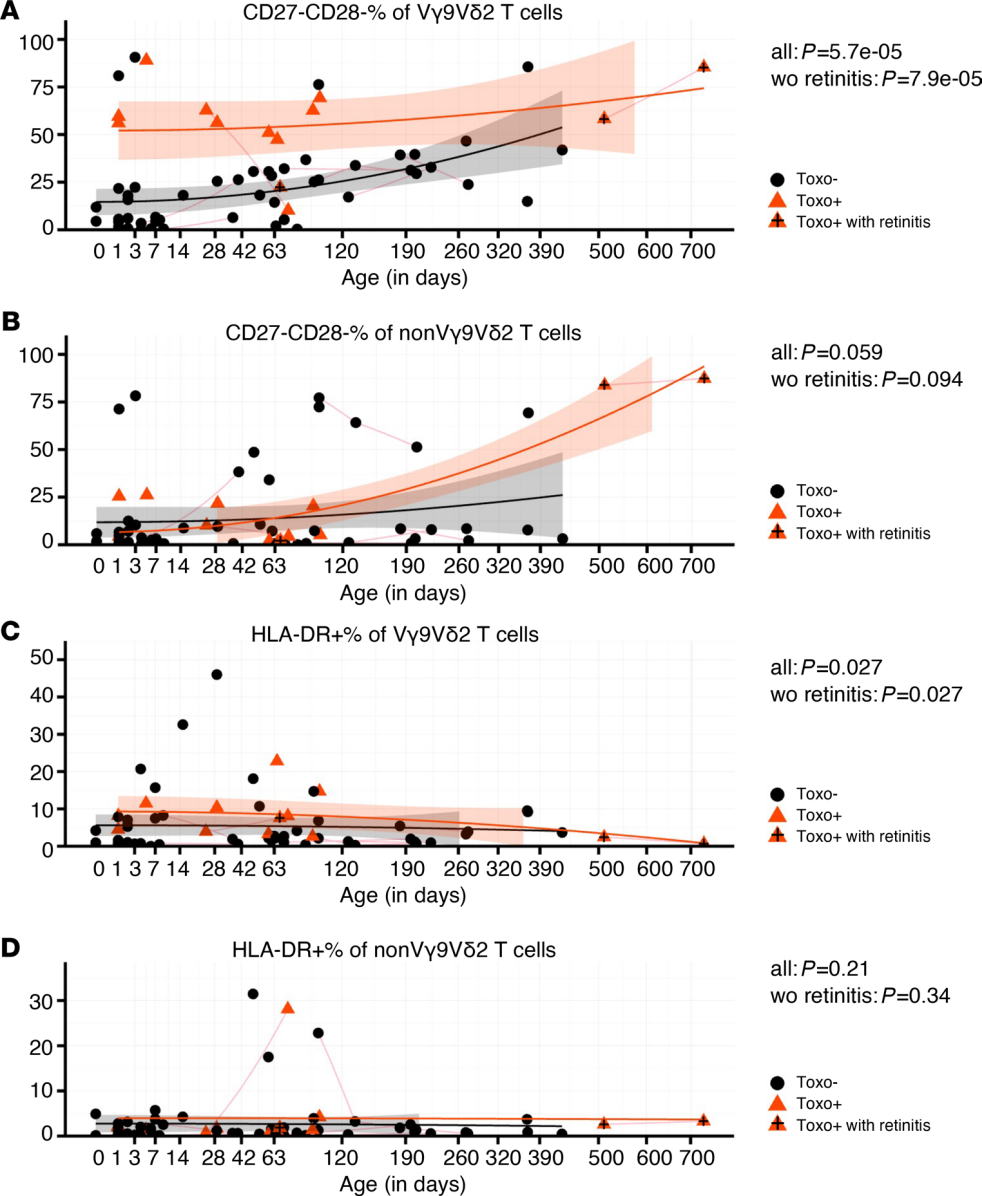
Vγ9Vδ2 T cells are differentiated upon congenital *T. gondii* infection. (**A**) Percentage of CD27^–^CD28^–^ cells of Vγ9^+^Vδ2^+^ γδ T cells versus age (sample number: Toxo^–^: *n* = 53, Toxo^+^: *n* = 13; subject number: *n* = 57, Toxo^+^ *n* = 11). (**B**) Percentage of CD27^–^CD28^–^ cells of non-Vγ9^+^Vδ2^+^ γδ T cells versus age (sample number: Toxo^–^: *n* = 53, Toxo^+^: *n* = 13; subject number: *n* = 57, Toxo^+^ *n* = 11). (**C**) Percentage of HLA-DR^+^ cells of Vγ9^+^Vδ2^+^ γδ T cells versus age (sample number: Toxo^–^: *n* = 53, Toxo^+^: *n* = 13; subject number: *n* = 57, Toxo^+^ *n* = 11). (**D**) Percentage of HLA-DR^+^ cells of non-Vγ9^+^Vδ2^+^ γδ T cells versus age (sample number: Toxo^–^: *n* = 53, Toxo^+^: *n* = 13; subject number: *n* = 57, Toxo^+^ *n* = 11). Toxo^+^ samples are indicated in orange triangles; Toxo^–^ samples are indicated in black dots. Lines connect samples of the same subject. Samples from subjects with symptoms (retinitis) are indicated with plus signs. *P* values of Toxo^+^ (with or without the subjects with symptoms) samples versus Toxo^–^ samples are calculated by Mann-Whitney *U* test. The 95% confidence interval (linear model) of more than 0 area is indicated for each group (orange for Toxo^+^, gray for Toxo^–^).

Next, we examined whether the expanded Vγ9Vδ2 T cells upon congenital toxoplasmosis were differentiated, as assessed by the downregulation of CD28 and CD27 (30–33). The Vγ9Vδ2 T cells of Toxo^+^ newborns were highly differentiated (CD27^–^CD28^–^) compared with Toxo^–^ newborns, while this was not the case for non-Vγ9Vδ2 γδ T cells (Figure 3B). The few Toxo^–^ newborns showing a CD28^–^CD27^–^ phenotype (Figure 3A) could be due to an early response to the postnatal exposure to the microbiome (34, 35), but an influence of exposure to *T*. *gondii*–derived metabolites in utero cannot be excluded (36). At later ages, the difference between the Toxo^+^ and Toxo^–^ groups became less pronounced due to an increase in differentiated Vγ9Vδ2 T cells in Toxo^–^ infants (Figure 3A). The level of HLA-DR expression, reflecting recent activation (26, 37), on Vγ9Vδ2 T cells, but not on non-Vγ9Vδ2 T cells, was also higher in the Toxo^+^ group (Figure 3, C and D), although this was more subtle compared with the differences observed regarding the differentiation status (compare Figure 3A with Figure 3C). Thus, it appears that the Vγ9Vδ2 T cells were activated and differentiated in utero during the strong expansion, after which proliferation and activation declined while the differentiation status remained stable and high until early after birth.

### Vγ9Vδ2 T cells express high levels of cytotoxic effector molecules upon congenital *T. gondii* infection.

**Figure 4:**
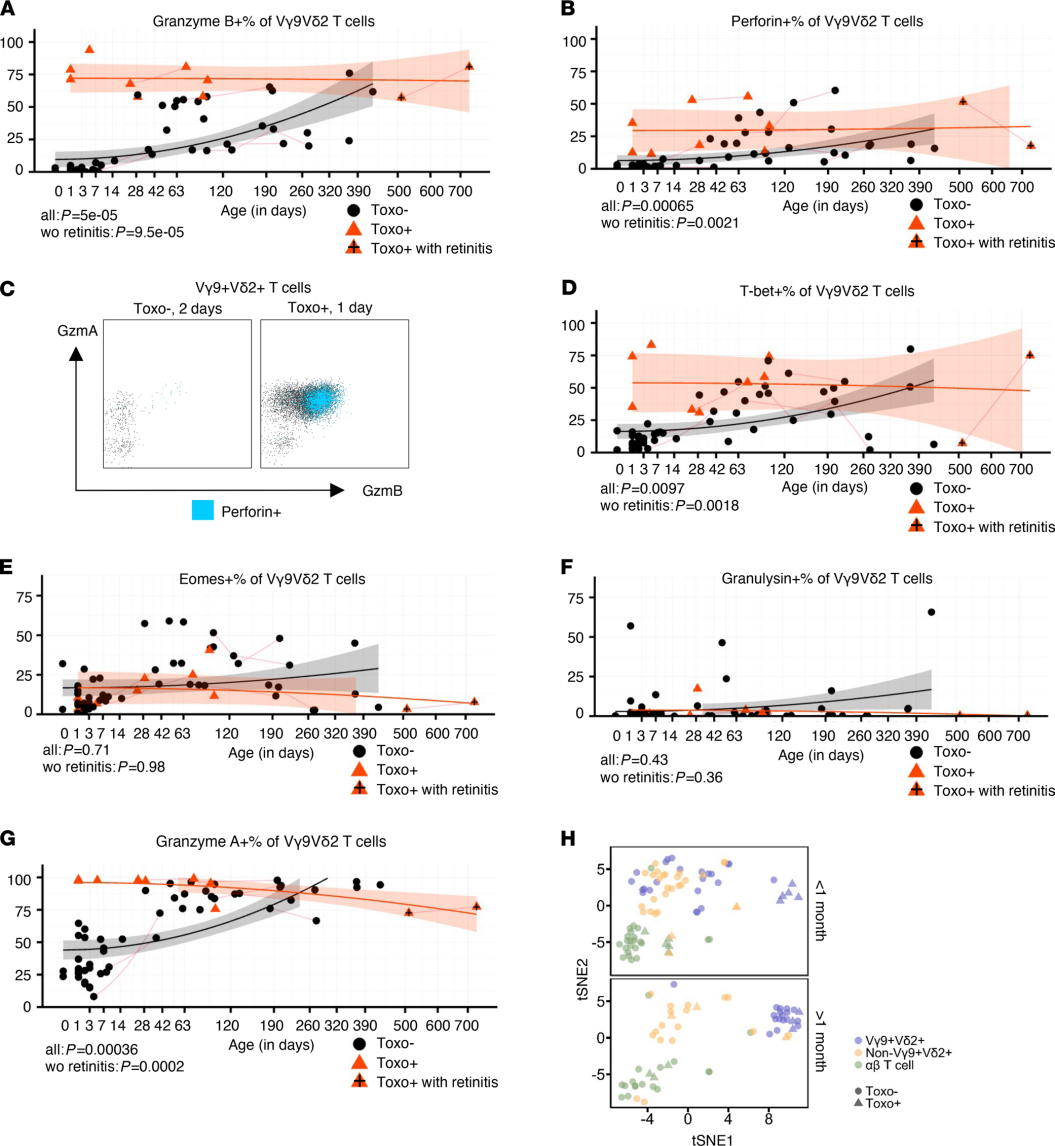
Vγ9Vδ2 T cells express high levels of cytotoxic effector molecules upon congenital *T. gondii* infection. (**A**) Percentage of GzmB^+^ cells of Vγ9^+^Vδ2^+^ γδ T cells versus age (sample: Toxo^–^: *n* = 53, Toxo^+^: *n* = 10; subject: *n* = 54, Toxo^+^ *n* = 8). (**B**) Percentage of perforin^+^ cells of Vγ9^+^Vδ2^+^ γδ T cells versus age (sample: Toxo^–^: *n* = 53, Toxo^+^: *n* = 10; subject: *n* = 54, Toxo^+^ *n* = 8). (**C**) Flow cytometry plots gated on Vγ9Vδ2 T cells of a representative sample of Toxo^–^ (2 days old) and Toxo^+^ (1 day old) newborns illustrating the expression of GzmA, GzmB, and perforin. (**D**) Percentage of T-bet^+^ cells of Vγ9^+^Vδ2^+^ γδ T cells versus age (sample: Toxo^–^: *n* = 55, Toxo^+^: *n* = 10; subject: *n* = 56, Toxo^+^ *n* = 8). (**E**) Percentage of eomes^+^ cells of Vγ9^+^Vδ2^+^ γδ T cells versus age (sample: Toxo^–^: *n* = 55, Toxo^+^: *n* = 10; subject: *n* = 56, Toxo^+^ *n* = 8). (**F**) Percentage of granulysin^+^ cells of Vγ9^+^Vδ2^+^ γδ T cells versus age (sample: Toxo^–^: *n* = 53, Toxo^+^: *n* = 10; subject: *n* = 54, Toxo^+^ *n* = 8). (**G**) Percentage of GzmA^+^ cells of Vγ9^+^Vδ2^+^ γδ T cells versus age (sample: Toxo^–^: *n* = 53, Toxo^+^: *n* = 10; subject: *n* = 54, Toxo^+^ *n* = 8). (**H**) t-SNE analysis of flow cytometry results (11 markers [HLA-DR, CD27, CD28, CD45RA, Ki-67, T-bet, eomes, GzmA, GzmB, granulysin, perforin], *n* = 48 subjects). Toxo^+^ samples are indicated in orange triangles; Toxo^–^ samples are indicated in black dots. Lines connect samples of the same subject. Samples from subjects with symptoms (retinitis) are indicated with plus signs. *P* values of Toxo^+^ (with or without the subjects with symptoms) samples versus Toxo^–^ samples are calculated by Mann-Whitney *U* test. The 95% confidence interval (linear model) of more than 0 area is indicated for each group (orange for Toxo^+^, gray for Toxo^–^).

We investigated whether the differentiation of the Vγ9Vδ2 T cells in utero was associated with the expression of cytotoxic effector molecules, which can be important for fighting infections (38). While fetal Vγ9Vδ2 T cells express granzyme A (GzmA) in the absence of infection, they do not express granzyme B (GzmB) or perforin (10), the main cytotoxic effector molecules that can efficiently kill infected cells (38). Indeed, newborn Vγ9Vδ2 T cells of the Toxo^–^ group did not express GzmB or perforin (Figure 4, A–C). However, upon congenital *T*. *gondii* infection, the expression of these two cytotoxic mediators strikingly increased: a vast majority of (newborn) Vγ9Vδ2 T cells expressed GzmB, while perforin was coexpressed in the GzmB^hi^ Vγ9Vδ2 T cells (Figure 4, A–C). The coexpression of GzmB and perforin is in line with the need of their combined actions to mediate their cytotoxic activity (38). At older ages, the Vγ9Vδ2 T cells of Toxo^–^ infants started to also express GzmB and perforin (Figure 4, A and B). A relatively small difference in GzmB^+^ and perforin^+^ non-Vγ9Vδ2 T cells could be observed between Toxo^+^ and Toxo^–^ subjects, possibly due to a bystander effect caused by cytokine production in the local environment (Supplemental Figure 4, A and B). Since the T-box family transcription factors T-bet and eomesodermin (eomes) can be important for the expression of GzmB and/or perforin (38), we investigated the expression of these transcription factors in Vγ9Vδ2 T cells. While the expression of T-bet followed the same expression pattern as GzmB and perforin (Figure 4D), this was not observed for eomes (Figure 4E). Granulysin is a cytotoxic granule pore-forming peptide that can permeabilize bacteria and parasites directly and deliver death-inducing GzmB into these pathogens (39). Furthermore, adult Vγ9Vδ2 T cells, which are expanded in the blood stage of malaria-infected patients (*Plasmodium falciparum*), are able to reduce parasite reinvasion in a granulysin-dependent manner (40, 41). However, in contrast to GzmB and perforin, granulysin remained low in Toxo^+^ infants, even at older ages (Figure 4F), indicating that this cytotoxic mediator does not play an important role in the defense of fetal Vγ9Vδ2 T cells against congenital *T*. *gondii* infection. Finally, we confirmed the programmed expression of GzmA (10) in Toxo^–^ newborns, which was further increased by congenital *T*. *gondii* infection (Figure 4G).

In order to have a global overview of all the flow cytometry data of Vγ9Vδ2 T cells in Toxo^+^ and Toxo^–^ infants and how they compare to the data obtained in non-Vγ9Vδ2 T cells and conventional αβ T cells, we performed t-distributed stochastic neighbor embedding (t-SNE) and principal components analysis (PCA). This analysis revealed that early after birth Vγ9Vδ2 T cells from Toxo^+^ infants were clearly forming a distinct cytotoxic effector-related cluster, while this was not the case for non-Vγ9Vδ2 T cells and αβ T cells (Figure 4H and Supplemental Figure 5). Later in life, the Vγ9Vδ2 T cells from Toxo^–^ and Toxo^+^ infants grouped together into 1 cluster (Figure 4H and Supplemental Figure 5). Thus, this global analysis highlights the early and potent response of Vγ9Vδ2 T cells toward congenital *T*. *gondii* infection, including the acquisition of a high coexpression of the cytotoxic effector molecules GzmB and perforin.

### The Vγ9Vδ2 TCR repertoire of Toxo^+^ infants contains a fetal footprint.

To address whether the observed expansion of Vγ9Vδ2 T cells upon congenital toxoplasmosis (Figures 1 and 2 and Supplemental Figures 1 and 2) shaped their TCR repertoire, we analyzed the CDR3 of the γ and δ chain of sorted blood γδ T cells of Toxo^+^ and Toxo^–^ infants at 2 months after birth (the earliest age at which we had a sufficient amount of sample material at the right conditions for TCR repertoire analysis; Toxo^+^ *n* = 5, Toxo^–^ *n* = 10) and at 1 year (Toxo^+^ *n* = 3, Toxo^–^ *n* = 4). At both age points, one of the Toxo^+^ infants had symptoms (retinitis).

**Figure 5:**
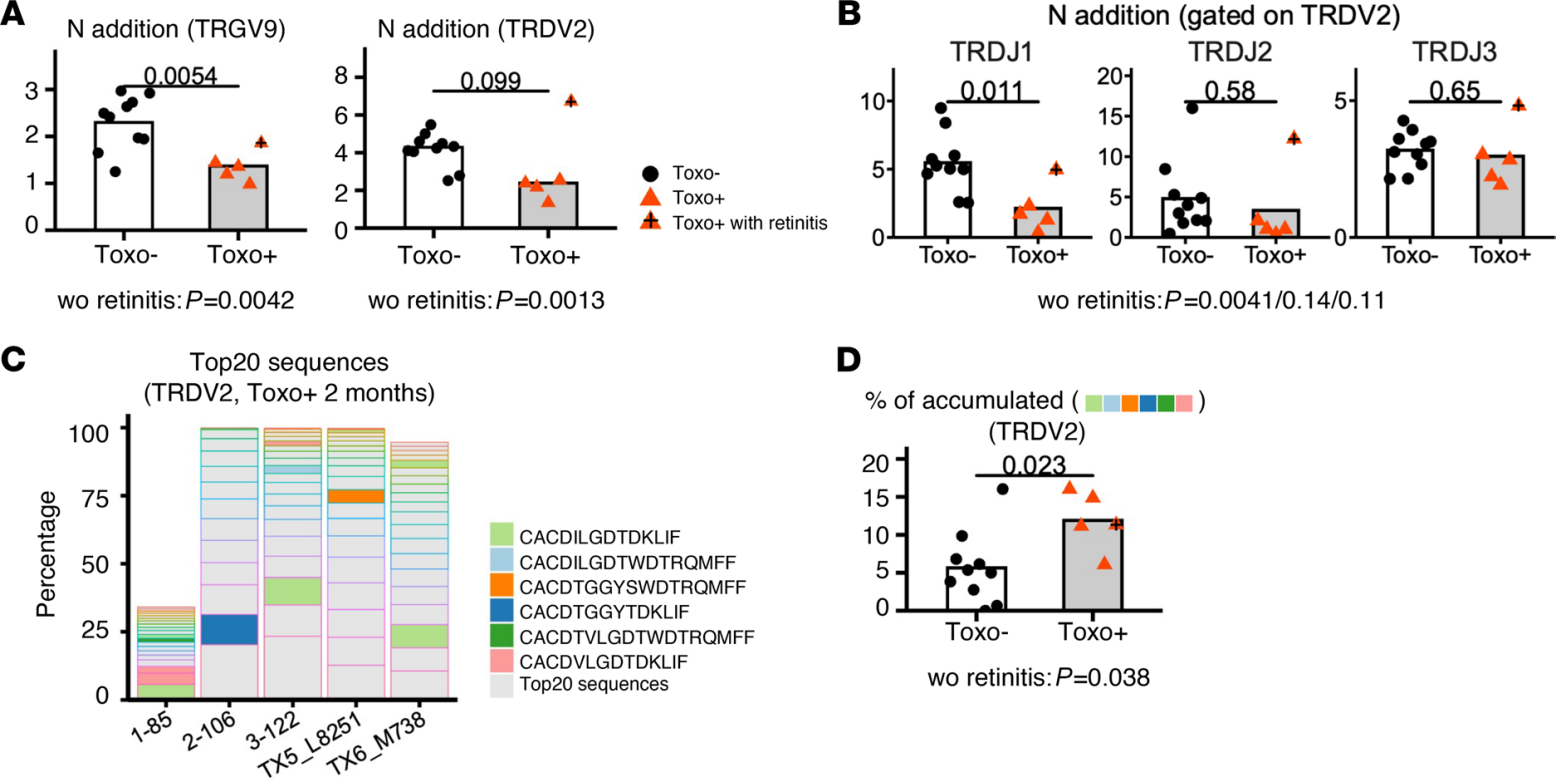
The Vγ9Vδ2 TCR repertoire of Toxo+ newborns contains a fetal footprint. (**A**) Number of N additions of TRGV9- and TRDV2-containing CDR3 of sorted blood γδ T cells from 2-month-old infants. *P* values (indicated on the bar graphs) are obtained by the Student’s *t* test for TRGV9 N addition (bar indicates mean) and by Mann-Whitney *U* test for TRDV2 N addition (bar indicates median). (**B**) Number of N additions of TRDV2-containing CDR3 using either TRDJ1, TRDJ2 or TRDJ3 of sorted blood γδ T cells from 2-month-old infants. *P* values (indicated on the bar graphs) are calculated by Student’s *t* test; bar indicates mean. (**C**) Accumulated percentage of the top 20 TRDV2-containing CDR3 sequences of 5 Toxo^+^ 2-month-old samples (obtained from sorted blood γδ T cells). Six germline-encoded sequences are indicated in different colors. (**D**) Accumulated percentage of the 6 germline-encoded sequences indicated in **C** in Toxo^+^ and Toxo^–^ 2-month-old infants (sorted blood γδ T cells). *P* value (indicated on the bar graph) is calculated by Student’s 2-tailed *t* test; bar indicates mean. One subject with symptoms (retinitis) is indicated. The *P* values obtained without this subject with symptom are indicated under each bar figure.

The random insertion of nucleotides (denoted by N) by the enzyme TdT into the junctions of the joining V(D)J gene segments can significantly increase the junctional diversity of the CDR3 region (42). The level of TdT expression is low in fetal life, which is associated with the absence or a low number of N additions in the CDR3 repertoire of fetal γδ T cells (17, 43). Compared with Toxo^–^ infants, the TRGV9- and TRDV2-containing CDR3 repertoire of Toxo^+^ infants at 2 months contained a lower number of N additions (Figure 5A), especially in the TRDV2-containing CDR3 sequences using TRDJ1 as a joining gene segment (Figure 5B). At 1 year, differences were less clear (Supplemental Figure 6, A and B). Note that the Toxo^+^ infant showing symptoms had the highest number of N additions, especially in the TRDV2-containing CDR3 (Figure 5, A and B). The difference in N additions between Toxo^+^ and Toxo^–^ infants was specific for the TRDV2- and TRGV9-containing sequences (Supplemental Figure 6C), which is in line with the distinct expansion of Vγ9Vδ2 T cells upon congenital *T*. *gondii* infection (Figure 2 and Supplemental Figures 1 and 2).

The lower number of N additions in the TRGV9- and TRDV2-containing TCR repertoire of 2-month-old Toxo^+^ infants (with the notable exception of the infant with symptoms) indicates a fetal origin since these features are known to be enriched in fetal blood Vγ9Vδ2 T cells (17). To address this more directly, we investigated the overlap of the TRGV9 and TRDV2 CDR3 infant repertoires with the repertoires derived from fetal blood (22–30 weeks gestation), cord blood (39–41 weeks gestation), and adult blood (26–64 years) (17) (Supplemental Figure 7). Compared with Toxo^–^ infants, the TRDV2 CDR3 repertoire of Toxo^+^ infants, when excluding the “outlier” infant with symptoms (Figure 5A, “N addition”) was shared more with the fetal blood repertoire (Supplemental Figure 7B, right panel). A tendency for such increased sharing was also observed when compared with term delivery cord blood but was completely absent when compared with adult blood (Supplemental Figure 7B, right panel). The TRGV9 repertoire did not show such differences in sharing between Toxo^+^ and Toxo^–^ infants (Supplemental Figure 7B, left panel), which is probably due to the high prevalence of the public clonotype CALWEVQELGKKIKVF (10, 27, 34) in both the Toxo^–^ and Toxo^+^ group (Supplemental Figure 6D). These data indicate that the Toxo^+^ TRDV2 repertoire at 2 months has an origin early in fetal life. Therefore, we verified the presence of early fetal germline-encoded (i.e., without N additions) TRDV2 CDR3 sequences (21, 34, 43) in the top 20 clonotypes of the Toxo^+^ infants (Figure 5C) and found that their accumulated frequency was more prevalent in 2-month Toxo^+^ compared with 2-month Toxo^–^ infants (Figure 5D). A possible explanation for the high prevalence of the CACDVLGDTDKLIF and CACDILGDTDKLIF TRDV2 CDR3 sequences in early fetal life (21, 34) is that the P nucleotide(s) needed to form these sequences can be derived from both the TRDV2 and the TRDD3 gene segment (Supplemental Figure 8). This can occur in an efficient way in the absence of N additions because of the low expression of the TdT enzyme in fetal life (43). In addition, there is a short-homology repeat present between the TRDD3 and TRDJ1 gene segments (Supplemental Figure 8). The prevalence of the third fetal liver sequence, CACDTGGYTDKLIF, can be explained by short-homology recombination (Supplemental Figure 8). Note that the short-homology repeat between TRDV2 and TRDD3 (the nucleotides *ac*) to form CACDTGGYTDKLIF has been described previously (43).

In summary, it appears that Vγ9Vδ2 clonotypes with a fetal origin expand extensively in utero when faced with congenital *T*. *gondii* infection, resulting in a TCR repertoire footprint that is still present at 2 months after birth.

## Discussion

Despite their high activation threshold in vitro, we show here that fetal Vγ9Vδ2 T cells can respond vigorously to a parasite infection in utero. A main finding of our study was the presence among congenital Toxo^+^ infants of a fetal footprint in their γδ TCR repertoire (including germline-encoded TCR sequences), most likely because of the high expansion of fetal Vγ9Vδ2 T cells in utero. In line with these in vivo observations, Vγ9Vδ2 T cell clones expressing fetal germline-encoded TCR sequences are responsive in vitro toward phosphoantigen-containing mycobacterial extracts (21). Moreover, cord blood Vγ9Vδ2 T cells proliferate upon incubation with *T*. *gondii*–infected PBMC in vitro (26). We propose that protection against phosphoantigen-generating pathogens, such as *T*. *gondii*, may have provided a selective pressure during evolution for the maintenance of the germline-encoded genetic elements needed for the generation of phosphoantigen-reactive TCRs early during fetal development (17, 19). This is in agreement with the high level of heritability of Vγ9Vδ2 T cells compared with other innate-like T cells (44). Vγ9Vδ2 T cells from the older infants of the Toxo^–^ group showed postnatal expansions, likely due to a more general phosphoantigen exposure (e.g., microbiome) (34, 35), thus reaching similar Vγ9Vδ2 T cell percentages as their Toxo^+^ counterparts. It is worth noting that the newborn who showed sequelae due to congenital *T*. *gondii* infection (retinitis) did not show a fetal footprint like the Toxo^+^ infants without symptoms. Further studies are needed to investigate whether the lack of a fetal Vγ9Vδ2 T cell footprint can be linked to the development of symptoms upon congenital *T*. *gondii* infection.

In contrast to our data, a previous study, mainly based on in vitro restimulation data, indicated that congenital *T*. *gondii* infection induces an anergic state in infant Vγ9Vδ2 T cells (45). However, other Vγ9Vδ2 T cell functions were not assessed and age-matched controls were lacking (10, 45–47). Our data indicate that a major wave of proliferation of fetal Vγ9Vδ2 T cells occurs in utero upon *T*. *gondii* encounter and is accompanied by the acquisition of the potent expression of cytotoxic mediators (GzmB^+^perforin^+^). These effector functions can be used to kill *T*. *gondii*–infected cells, as illustrated by the in vitro study of Subauste et al. with Vγ9Vδ2 T cell lines and clones (26). Such a response that combines innate (germline-encoded TCR acting as a pathogen recognition receptor) and adaptive (high proliferation upon pathogen encounter) features has been referred to as “adaptate” biology (48). Hara et al. suggested that Vγ9Vδ2 T cells are susceptible to anergy induction because of their extrathymic development (45). However, we have recently shown that the human thymus clearly contains Vγ9^+^Vδ2^+^ cells (17). Based on our data from the current study, we conclude that congenital *T*. *gondii* infection does not induce an anergic state of fetal Vγ9Vδ2 T cells but rather transforms them to lymphocytes with a potent cytotoxic phenotype that contributes to protection against infection by killing *T*. *gondii*–infected cells.

We have previously shown that fetal non-Vγ9Vδ2 γδ T cells, such as the public Vγ8Vδ1 T cells, play a major role in the response toward congenital human cytomegalovirus (HCMV) infection (30). Together with the data of our current study, it appears that the human fetus is equipped with γδ T cell subsets that show a division of labor in their response to congenital infections: (i) the Vγ9Vδ2 T cells that respond to *T*. *gondii* and possibly other phosphoantigen-generating pathogens and (ii) the non-Vγ9Vδ2 T cells that target HCMV-infected cells. Data from human in vitro studies (26, 30) and in vivo studies in mice (25, 49, 50) indicate that γδ T cells play a protective role against infections with *T*. *gondii* and HCMV, but it cannot be excluded that the potent effector γδ T cells contribute to the development of pathologies observed upon congenital infections (51, 52).

A main correlate of protection of the malaria vaccine PfSPZ (attenuated *Plasmodium falciparum* sporozoite) are Vγ9Vδ2 T cells (53, 54). Our data, showing the importance of Vγ9Vδ2 T cells in the response toward congenital *T*. *gondii* infection, indicate that vaccines, or other strategies, could be developed targeting these cells to protect infants against (congenital *T*. *gondii*) infections. Tools to manipulate Vγ9Vδ2 T cells in vivo are becoming increasingly available and include modified phosphoantigens with improved pharmacological characteristics and monoclonal antibodies targeting BTN3A1 (55, 56). Both *Plasmodium falciparum* and *T*. *gondii* contain an organelle, the apicoplast, which has specific metabolic functions including the MEP pathway of isoprenoid synthesis. In this pathway, the metabolite HMBPP, the most potent natural phosphoantigen, is generated (4, 57). This indicates that *T*. *gondii*–derived HMBPP is a major driving force for the expansion of fetal Vγ9Vδ2 T cells in utero. However, in contrast to our observations in congenital toxoplasmosis, Cairo et al. observed a depletion of phosphoantigen-reactive Vγ9Vδ2 T cells in placental malaria (27). A main difference between congenital *T*. *gondii* infection and placental malaria is that the malaria parasite very rarely crosses the placenta into the fetal circulation to establish an infection (58). Furthermore, the type of placental malaria infection can have opposing effects on the immune system in early life, thus possibly contributing to the differential effect on the fetal Vγ9Vδ2 T cells (27, 58).

In immunocompromised (adult) patients (AIDS and transplant patients), toxoplasmosis is a major cause of morbidity and mortality (23). HIV infection leads to a decrease of Vγ9Vδ2 T cells (59), but it is not clear to what extent the depletion of these potential *T*. *gondii*–responsive cells contributes to *T*. *gondii*–induced morbidities. HCMV infection is a major driving force of non-Vγ9Vδ2 γδ T cell expansion in organ transplant and hematopoietic stem cell transplant patients (60–62), and these expansions are associated with reduced cancer development (63, 64). In contrast, the role of *T*. *gondii* infection in driving Vγ9Vδ2 T cell expansion/differentiation in these transplant settings and their potential anticancer role is not known. Therefore, the role of Vγ9Vδ2 T cells in toxoplasmosis in transplant and AIDS patients deserves further investigation.

Overall, our data indicate that the human fetus, from early gestation onward, possesses Vγ9Vδ2 T cells that can expand and transform into killer effector cells upon congenital *T*. *gondii* infection. Thus, these fetal innate T cells could provide protection against parasite infections in utero.

## Methods

### Blood sample collection and processing.

Peripheral blood was collected in the Microbiology and Virology outpatient center of IRCCS San Matteo Hospital Foundation, Pavia, Italy. The diagnosis of congenital toxoplasmosis was performed with Liaison XL Toxo IgG II / IgM CLIA, Novalisa IgA (DiaSorin), VIDAS Toxo IgG II, ISAGA IgM (bioMérieux), IgG-IgM Western blot (LDBio), and homemade interferon-γ release assay (65). IGRA test was performed in the same way as the test developed by Chapey and colleagues (65) with some modification, i.e., the antigen employed (the same antigen utilized for Liaison commercial tests) with a final concentration of 3 μg/mL, which was provided by DiaSorin. This concentration yielded the best results according to previous studies. The ELISA test to evaluate interferon-γ production is a commercially available kit (QIAGEN).

All the mothers (recruited from Italy, *n* = 72) from both infected and noninfected groups were diagnosed with *T*. *gondii* infection during pregnancy and were treated in the same way (with spyramicin and/or pyrimethamine + sulfodiazine + folinic acid) (66). Depending on the volume of collected blood, samples were either directly lysed (~0.5 mL) with FACS-lysing solution (BD FACS Lysing Solution) or processed (~1 mL) to isolate PBMCs with Lymphoprep gradient centrifugation (Lymphoprep, STEMCELL Technologies) and stored in liquid nitrogen. Frozen PBMC samples and FACS-lysed blood samples were then sent to the Institute for Medical Immunology of the ULB in Belgium.

### Flow cytometry and antibody reagents.

FACS-lysed samples were thawed from liquid nitrogen at 37°C and washed with PBS containing 0.1% bovine serum albumin (BSA) (MilliporeSigma). For surface staining, cells were incubated with antibody mix at 4°C for 15–20 minutes, then washed and resuspended with 0.1% BSA/PBS. For intracellular staining, after surface staining, the Perm 2 kit (BD Biosciences) was used to permeabilize cell membranes. All samples were acquired either on CyAn ADP cytometer (Dako Cytomation) or LSRFortessa (BD Biosciences); analysis was done using FlowJo software and R.

The following antibodies were used in this study: CD3-PB (clone SP34-2, BD Biosciences), CD3-BV510 (UCHT1, BD Biosciences), TCR γδ-APC (11F2, Miltenyi Biotec), TCR Vγ9-PC5 (IMMU 360, Beckman Coulter), TCR Vδ2-FITC (IMMU 389, Beckman Coulter), CD27-PE (M-T271, BD Biosciences), CD28-ECD (CD28.2, Beckman Coulter), CD45RA-PC7 (L48, BD Biosciences), HLA-DR-V450 (G46-6, BD Biosciences), Ki-67-PC7 (B56, BD Biosciences), T-bet-BV421 (4B10, BioLegend), eomes-PE (WD1928, eBioscience, Thermo Fisher Scientific), granzyme A-PB (CB9, BioLegend), granzyme B-PE-CF594 (GB11, BD Biosciences), granulysin-PE (eBioDH2 [DH2], Invitrogen, Thermo Fisher Scientific), and perforin-PC7 (dG9, delta G9; eBioscience, Thermo Fisher Scientific).

### Cell sorting, RNA isolation, and CDR3 analysis.

For PBMC samples, cells were thawed at 37°C in complete medium [RPMI 1640 from Gibco, Thermo Fisher Scientific, supplemented with l-glutamine (2 mM), penicillin (50 U/mL), streptomycin (50 U/mL), and 1% nonessential amino acids from Lonza and 10% (v/v) heat-inactivated FCS from PPA Laboratories], then labeled with Zombie NIR dye (BioLegend) at room temperature for 10 minutes and stained with CD3/TCR γδ/TCR Vγ9/TCR Vδ2 antibodies at 4°C for 15 minutes. CD3^+^TCR γδ^+^ T cells were sorted on FACSAria III (BD Biosciences) with a mean purity of 98.0%. Cells were snap-frozen in liquid nitrogen and preserved in –80°C.

RNA was isolated from sorted γδ T cells (~10,000 cells) with the RNeasy Micro Kit (QIAGEN). cDNA was generated by performing a template-switch anchored reverse transcription PCR. RNA was reverse-transcribed via a template-switch cDNA reaction using TRGC-specific (5′-CAAGAAGACAAAGGTATGTTCCAG-3′) and TRDC-specific (5′-GTAGAATTCCTTCACCAGACAAG-3′) primers in the same reaction tube, a template-switch adapter (5′-AAGCAGTGGTATCAACGCAGAGTACATrGrGrG-3′), and the Superscript II RT enzyme (Invitrogen, Thermo Fisher Scientific). The TRGC primer binds both TRGC1 and TRGC2. The cDNA was then purified using AMPure XP Beads (Agencourt). Amplification of the TRG and TRD region was achieved using a specific TRGC primer (binding both TRGC1 and TRGC2 5′-GTCTCGTGGGCTCGGAGATGTGTATAAGAGACAGAATAGTGGGCTTGGGGGAAACATCTGCAT-3′, adapter underlined) and a specific TRDC primer (5′-GTCTCGTGGGCTCGGAGATGTGTATAAGAGACAGACGGATGGTTTGGTATGAGGCTGACTTCT-3′, adapter underlined) and a primer complementary to the template-switch adapter (5′-TCGTCGGCAGCGTCAGATGTGTATAAGAGACAGAAGCAGTGGTATCAACGCA G-3′, adapter underlined) with the KAPA Real-Time Library Amplification Kit (Kapa Biosystems). Adapters were required for subsequent sequencing reactions. After purification with AMPure XP beads, an index PCR with Illumina sequencing adapters was performed using the Nextera XT Index Kit. This second PCR product was again purified with AMPure XP beads. High-throughput sequencing of the generated amplicon products containing the TRG and TRD sequences was performed on an Illumina MiSeq platform using the V2 300 kit, with 150 base pairs (bp) at the 3′ end (read 2) and 150 bp at the 5′ end (read 1) (at the GIGA center, University of Liège, Belgium).

After passing the quality check using fastqc (version 0.11.8, http://www.bioinformatics.babraham.ac.uk/projects/fastqc/), raw sequencing reads from fastq files (read 1 and read 2) were aligned to reference V, D, and J genes from GenBank database specifically for “TRG” or “TRD” to build CDR3 sequences using the MiXCR software (version 3.0.3) (67). Default parameters were used except to assemble the TRDD gene segments where 3 instead of 5 consecutive nucleotides were applied as the assembly parameter. CDR3 sequences were then exported and analyzed using VDJtools software (version 1.2.1) using default settings in order to calculate the number of N additions, the CDR3 length, the length of J gene segments, and the level of clonotype sharing between different samples (68). Sequences out of frame and containing stop codons were excluded from the analysis. Files generated from VDJtools were uploaded into Rstudio (version 1.1.463, R version 3.5.2), and analysis involved the following packages: ggplot2, dplyr, reshape, ggpubr, and ggseqlogo. Fastq files of TRG and TRD sequences are deposited in the Sequence Read Archive under accession number PRJNA625515.

### Dimensionality reduction and clustering.

Flow cytometry results generated from FlowJo and CDR3 data generated from VDJtools were uploaded into Rstudio. Packages ggfortify (https://CRAN.R-project.org/package=ggfortify) and ggbiplot (http://github.com/vqv/ggbiplot) were used to generate PCA. Package Rtsne (https://github.com/jkrijthe/Rtsne) was used to generate t-SNE clustering analysis (69). Parameters were adapted according to sample size. t-SNE analyses were run multiple times using different parameters.

### Statistics.

Statistical analysis was done by using R. Student’s 2-tailed *t* test was used for normally distributed data (Shapiro-Wilk test, *P* > 0.05) and with equal variances (Levene’s test, *P* > 0.05). Otherwise, Mann-Whitney *U* test was used. When more than 1 blood sample was obtained from the same subject at different ages (e.g., Figure 1: in 8 out of 74 infants), only the earliest sample was used from this subject in the statistical test for the calculation of the *P* value between Toxo^+^ and Toxo^–^ infants. For analysis of the data according to age, the linear model was used for prediction; the point-wise 95% confidence interval was indicated around the mean.

### Study approval.

This study was approved by IRCCS San Matteo Polyclinic Foundation ethical committee number 20160017812. All parents were provided with written and oral information about the study and gave their consent. Research was conducted in accordance with the Declaration of Helsinki.

## Author contributions

LM, MP, and MT conducted experiments; FG and VM acquired crucial blood samples; AM, VM, and DV designed the study; LM and DV analyzed data; and DV wrote the manuscript.

## Supplementary Material

Supplemental data

## Figures and Tables

**Table 1:**
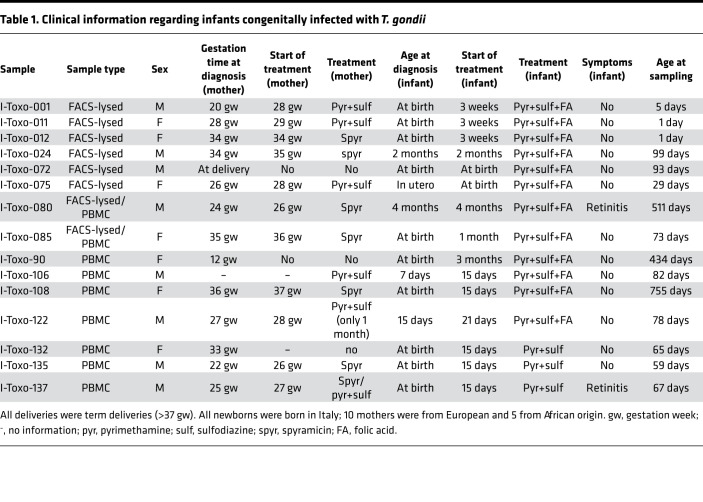
Clinical information regarding infants congenitally infected with *T. gondii*

## Version 1 07/13/2021

In-Press Preview

## Version 2 08/23/2021

Electronic publication
