# Supplementary material for: Effector Vγ9Vδ2 T cell response to congenital Toxoplasma gondii infection
Source: JCI Insight. 2021 Aug 23;6(16):e138066. doi: 10.1172/jci.insight.138066 (PMC8409983; doi:10.1172/jci.insight.138066)
Supplement: Supplemental data [file jciinsight-6-138066-s094.pdf]

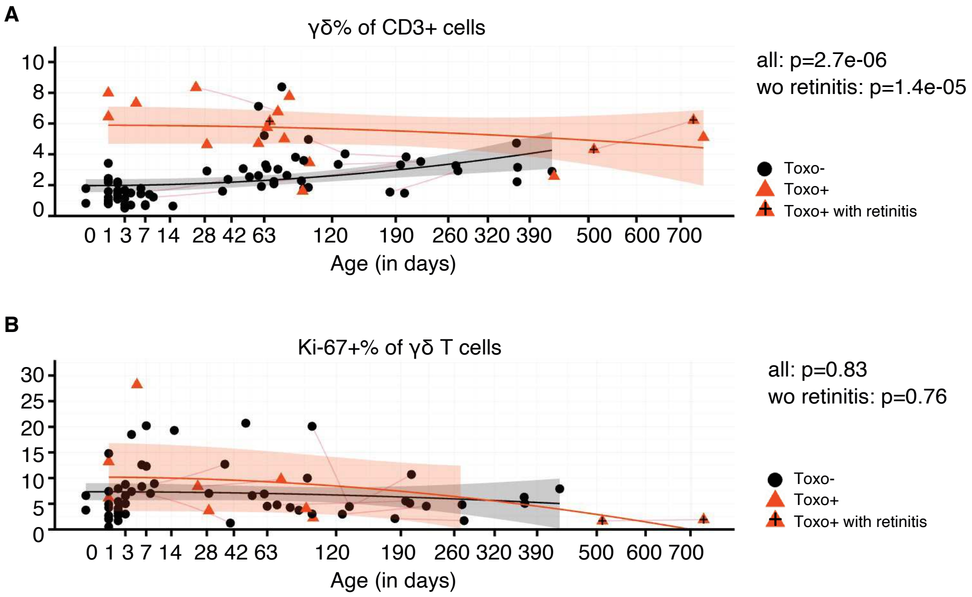
A
γδ% of CD3+ cells
all: p=2.7e-06
wo retinitis: p=1.4e-05
Toxo-
Toxo+
Toxo+ with retinitis
0 1 3 7 14 28 42 63 120 190 260 320 390 500 600 700
Age (in days)
B
Ki-67+% of γδ T cells
all: p=0.83
wo retinitis: p=0.76
Toxo-
Toxo+
Toxo+ with retinitis
0 1 3 7 14 28 42 63 120 190 260 320 390 500 600 700
Age (in days)

Figure 1

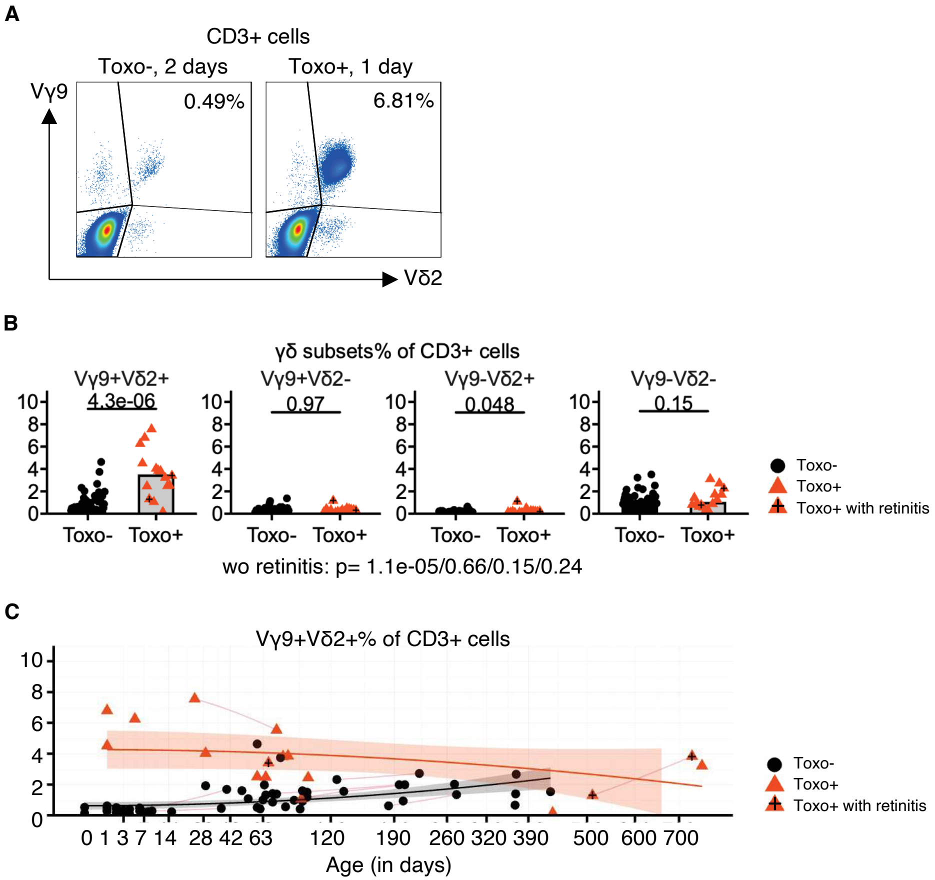
A
CD3+ cells
Toxo-, 2 days
Toxo+, 1 day
Vγ9
0.49%
6.81%
Vδ2
B
γδ subsets% of CD3+ cells
Vγ9+Vδ2+
4.3e-06
Vγ9+Vδ2-
0.97
Vγ9-Vδ2+
0.048
Vγ9-Vδ2-
0.15
Toxo-
Toxo+
Toxo+ with retinitis
wo retinitis: p= 1.1e-05/0.66/0.15/0.24
C
Vγ9+Vδ2+% of CD3+ cells
0 1 3 7 14 28 42 63 120 190 260 320 390 500 600 700
Age (in days)

Figure 2

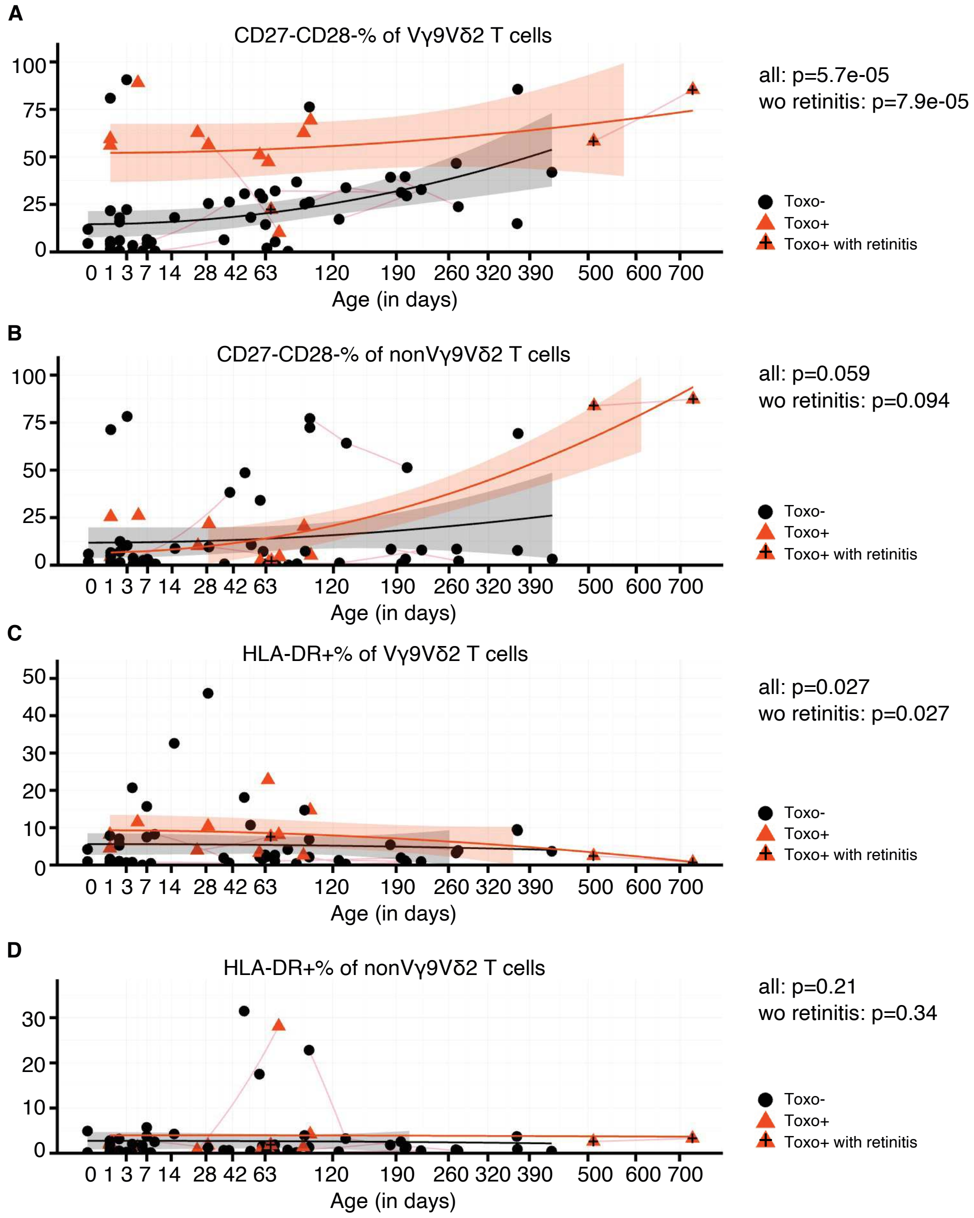
A
CD27-CD28-% of Vγ9Vδ2 T cells
all: p=5.7e-05
wo retinitis: p=7.9e-05
Toxo-
Toxo+
Toxo+ with retinitis
Age (in days)
B
CD27-CD28-% of nonVγ9Vδ2 T cells
all: p=0.059
wo retinitis: p=0.094
C
HLA-DR+% of Vγ9Vδ2 T cells
all: p=0.027
wo retinitis: p=0.027
D
HLA-DR+% of nonVγ9Vδ2 T cells
all: p=0.21
wo retinitis: p=0.34

Figure 3

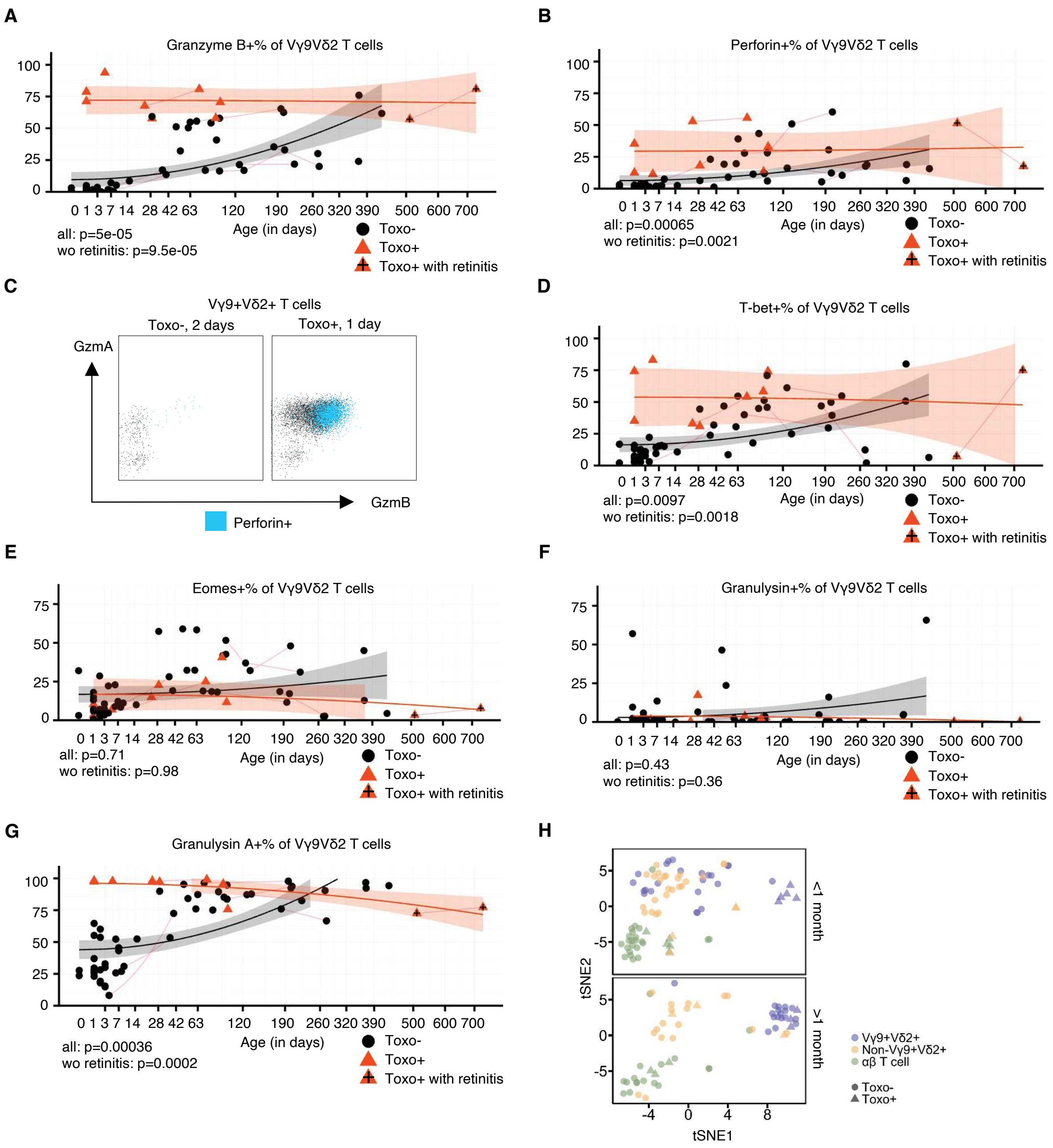

A
Granzyme B+% of Vγ9Vδ2 T cells
all: p=5e-05
wo retinitis: p=9.5e-05
Age (in days)
Toxo-
Toxo+
Toxo+ with retinitis
B
Perforin+% of Vγ9Vδ2 T cells
all: p=0.00065
wo retinitis: p=0.0021
C
Vγ9+Vδ2+ T cells
Toxo-, 2 days
Toxo+, 1 day
GzmA
GzmB
Perforin+
D
T-bet+% of Vγ9Vδ2 T cells
all: p=0.0097
wo retinitis: p=0.0018
E
Eomes+% of Vγ9Vδ2 T cells
all: p=0.71
wo retinitis: p=0.98
F
Granulysin+% of Vγ9Vδ2 T cells
all: p=0.43
wo retinitis: p=0.36
G
Granulysin A+% of Vγ9Vδ2 T cells
all: p=0.00036
wo retinitis: p=0.0002
H
tSNE1
tSNE2
<1 month
>1 month
Vγ9+Vδ2+
Non-Vγ9+Vδ2+
αβ T cell
Toxo-
Toxo+

Figure 4

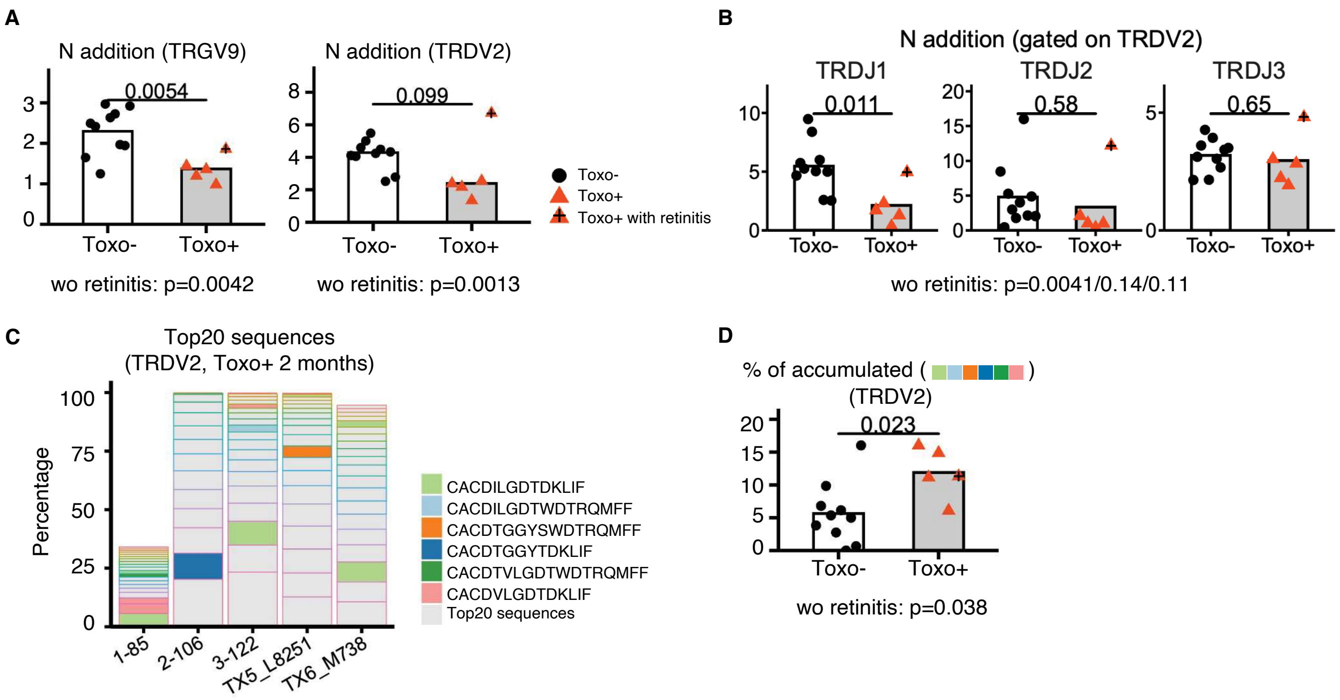
A
N addition (TRGV9)
0.0054
Toxo-
Toxo+
wo retinitis: p=0.0042
N addition (TRDV2)
0.099
wo retinitis: p=0.0013
Toxo-
Toxo+
Toxo+ with retinitis
B
N addition (gated on TRDV2)
TRDJ1
0.011
TRDJ2
0.58
TRDJ3
0.65
wo retinitis: p=0.0041/0.14/0.11
C
Top20 sequences
(TRDV2, Toxo+ 2 months)
Percentage
CACDILGDTDKLIF
CACDILGDTWDTRQMFF
CACDTGGYSWDTRQMFF
CACDTGGYTDKLIF
CACDTVLGDTWDTRQMFF
CACDVLGDTDKLIF
Top20 sequences
1-85
2-106
3-122
TX5_L8251
TX6_M738
D
% of accumulated ( )
(TRDV2)
0.023
Toxo-
Toxo+
wo retinitis: p=0.038

Figure 5

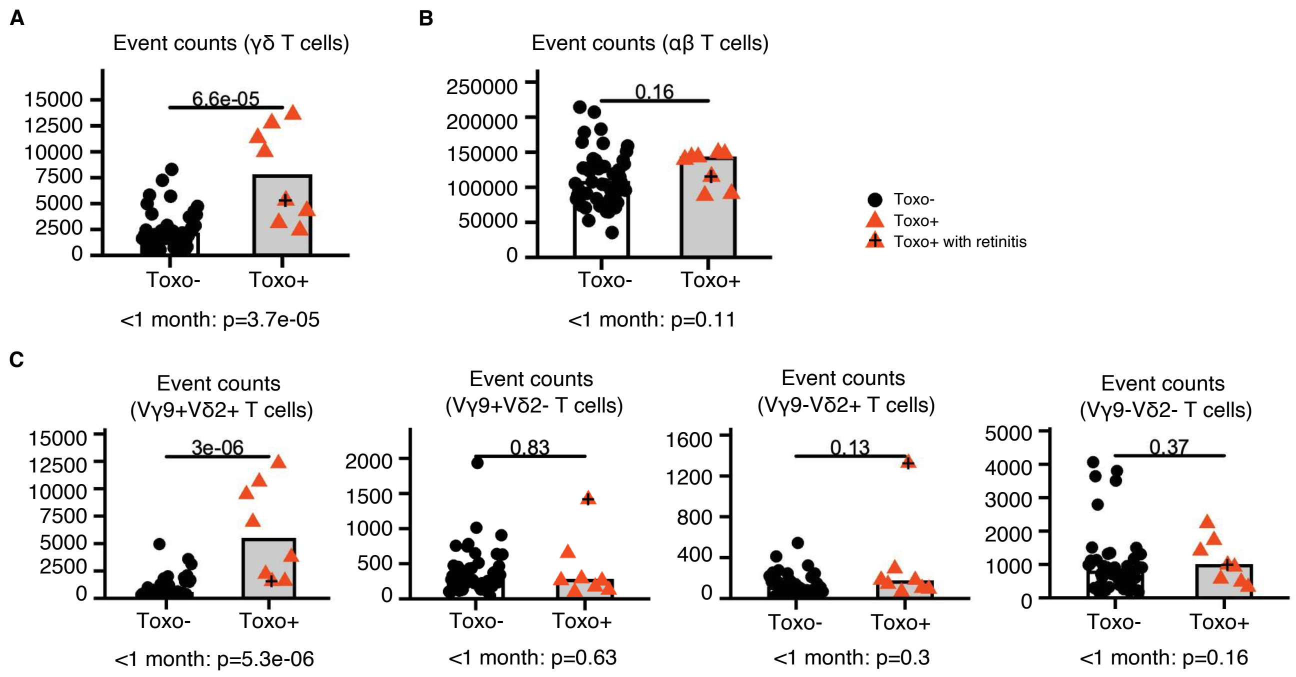

Supplemental figure 1

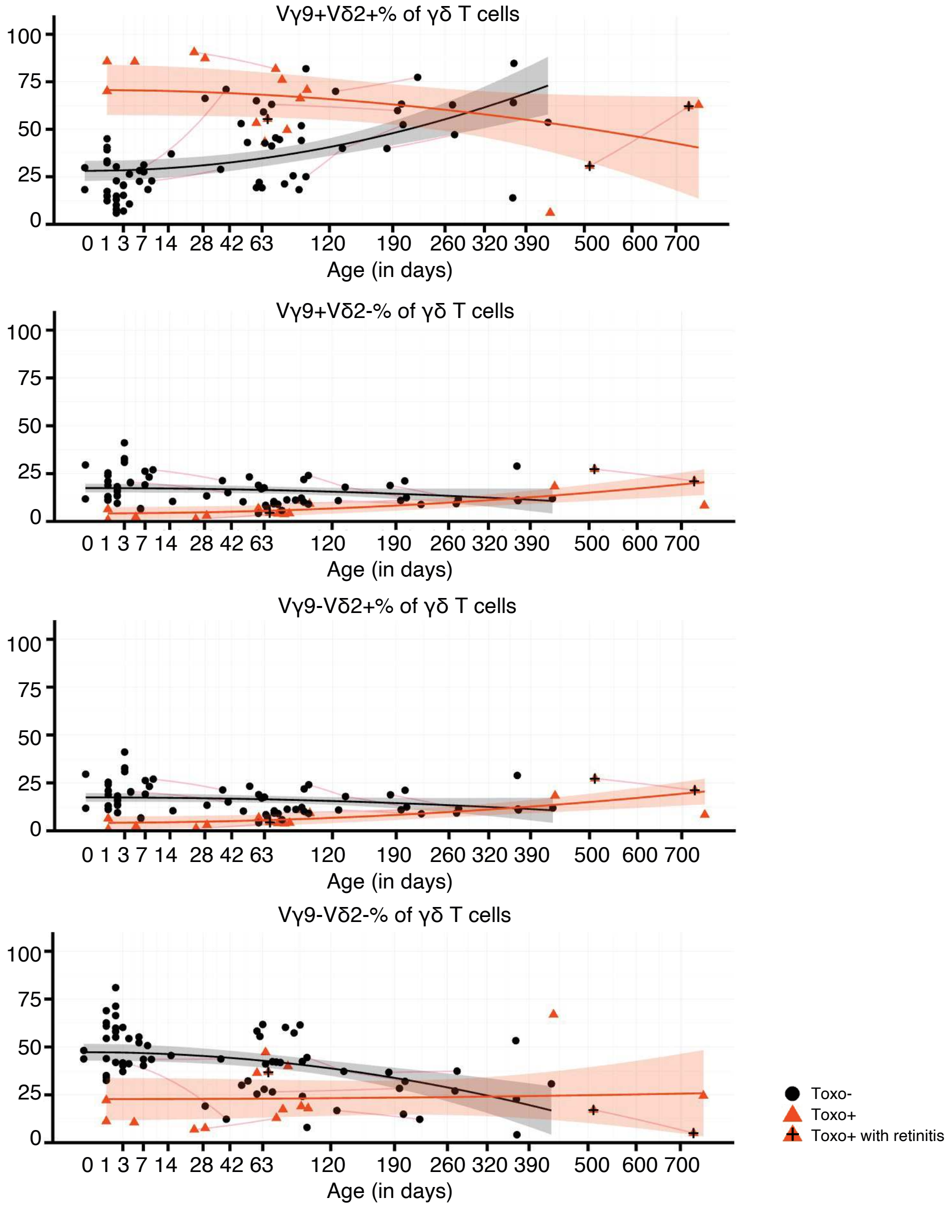

Supplemental figure 2

A

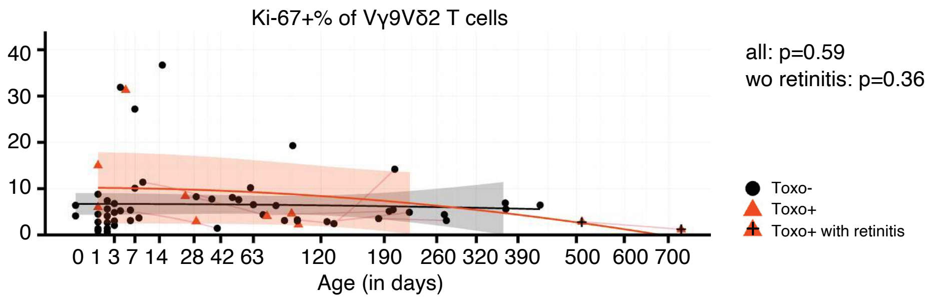

B

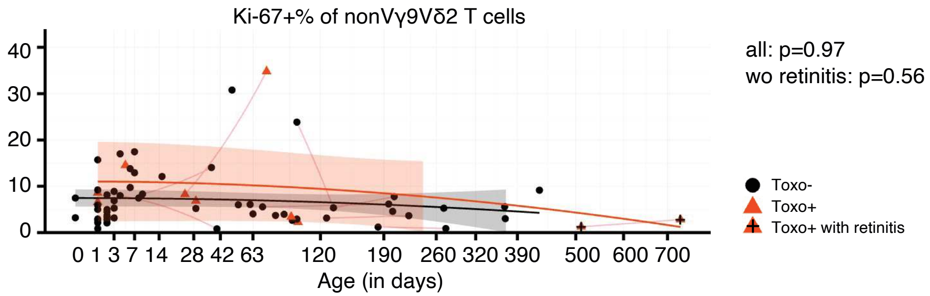

Supplemental figure 3

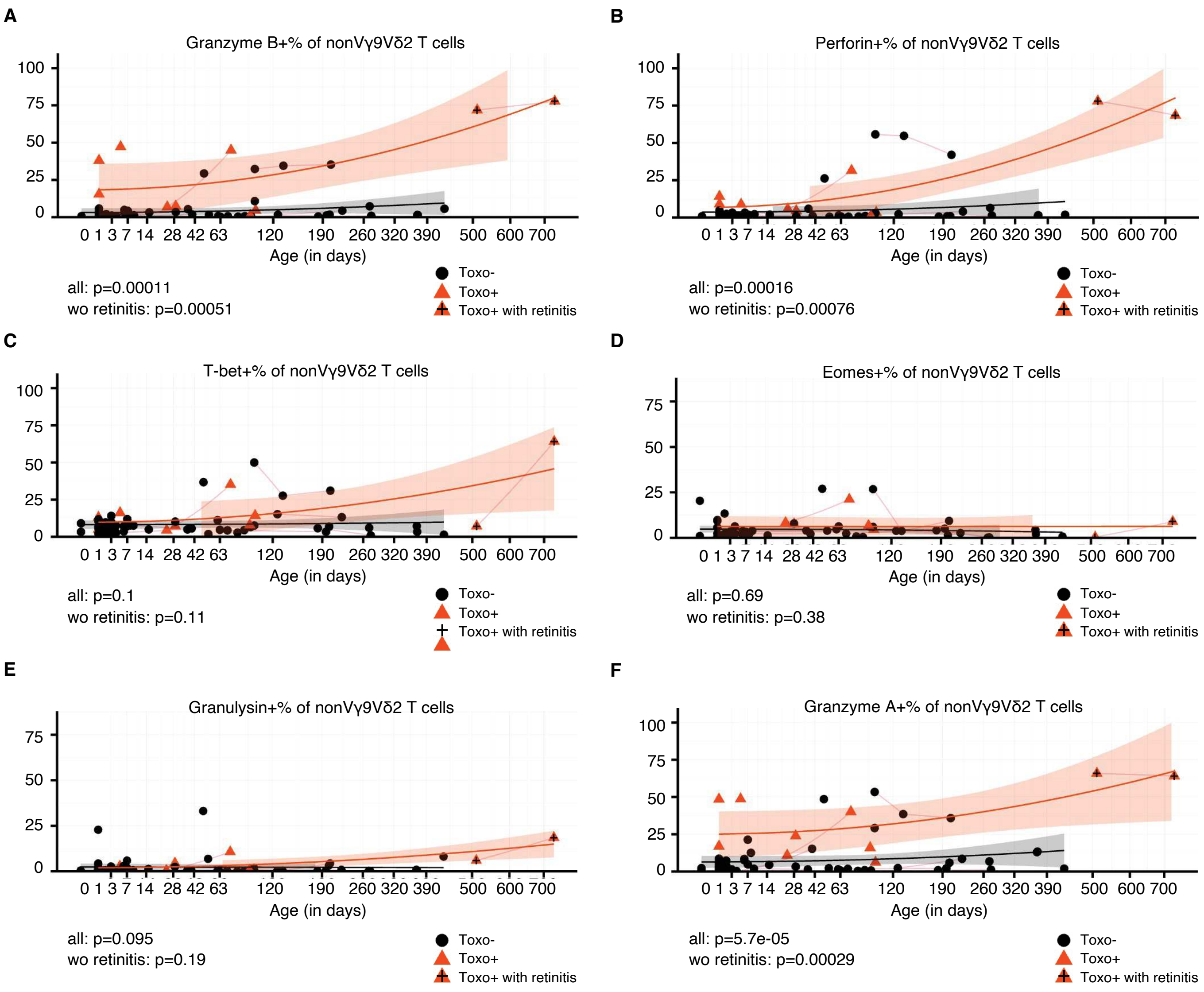

Supplemental figure 4

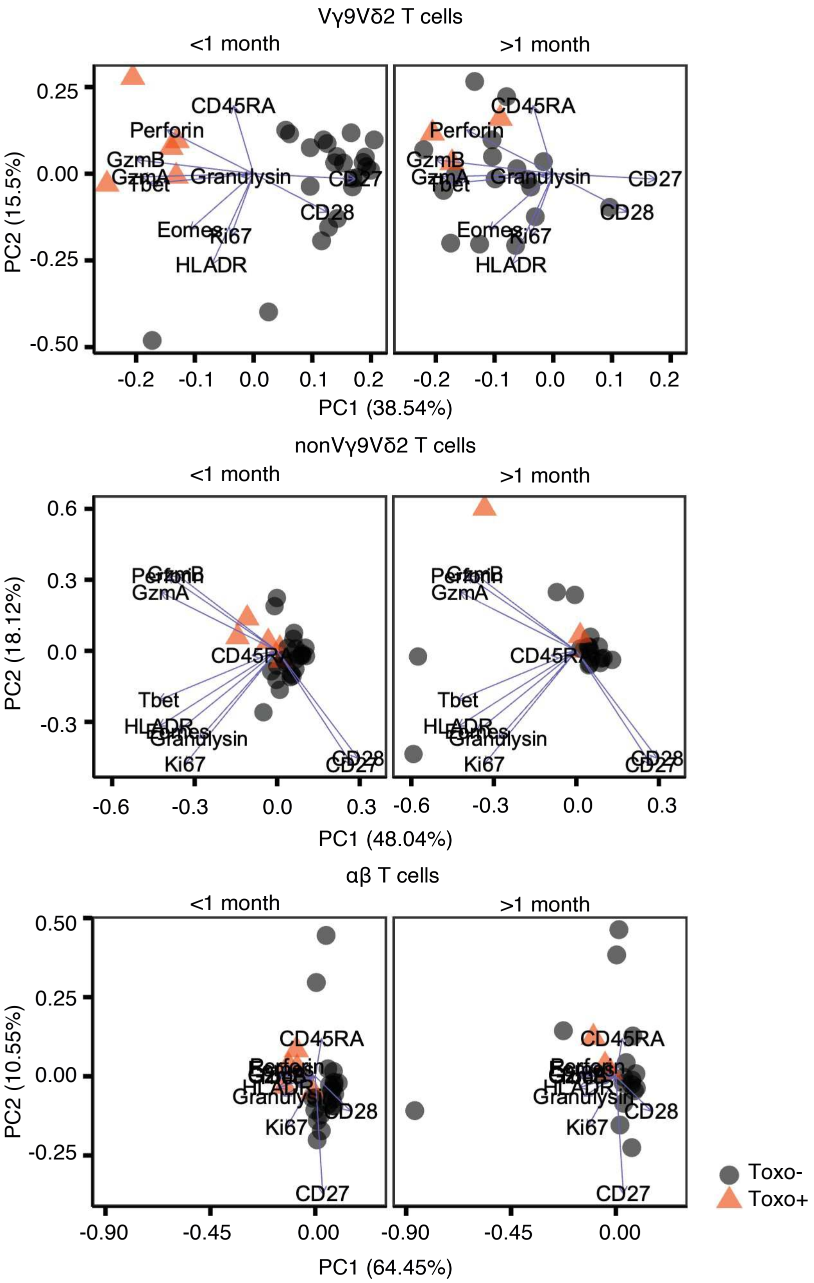

Supplemental figure 5

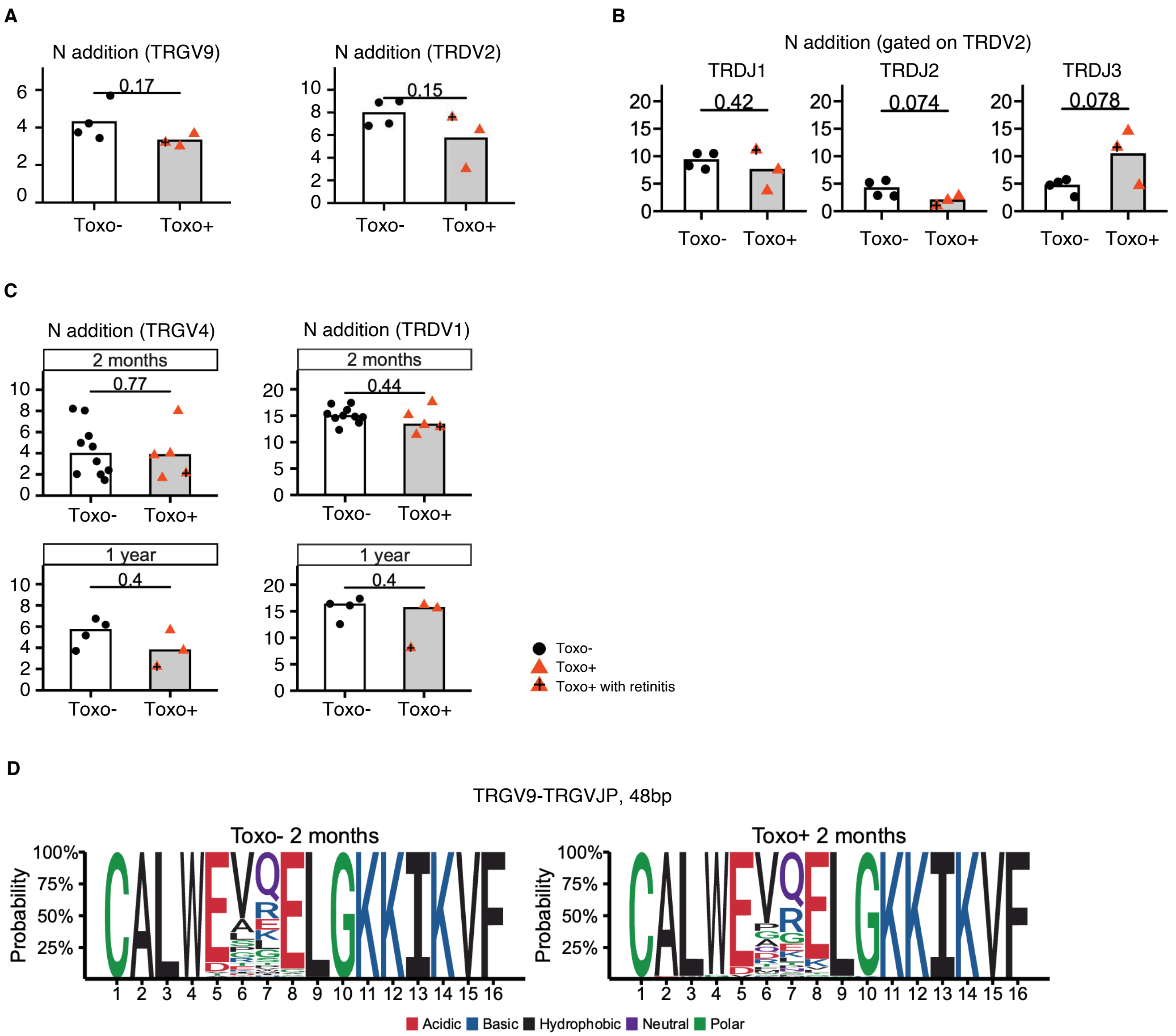

Supplemental figure 6

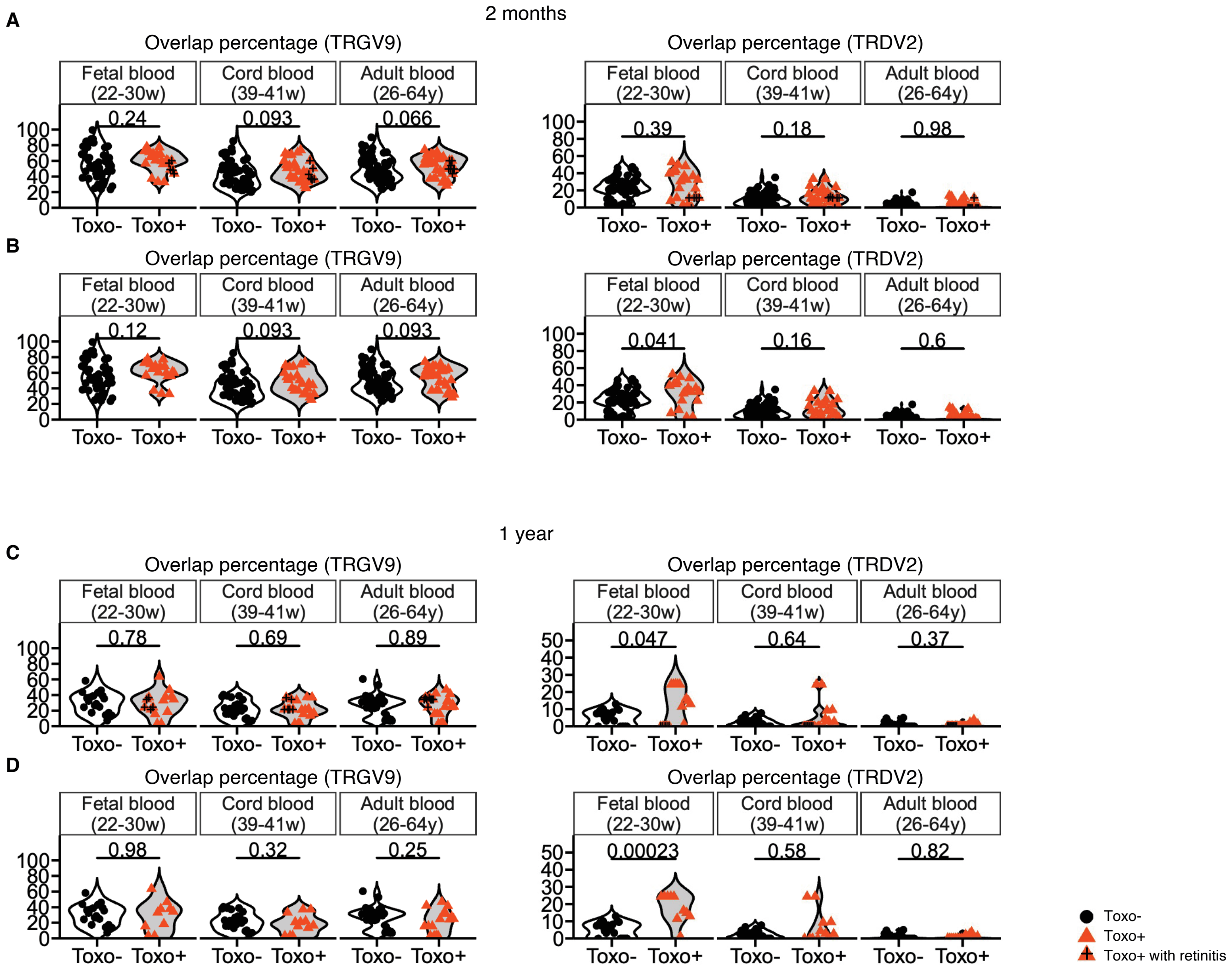

Supplemental figure 7

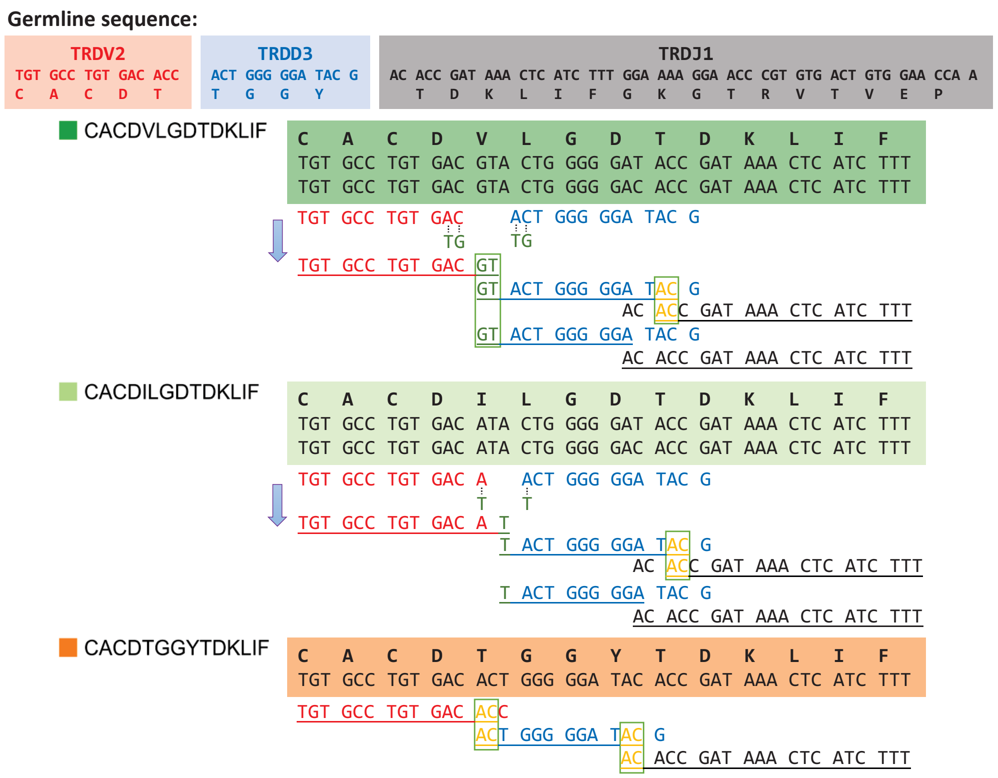

Supplemental figure 8
